# Maternal serum uric acid level and maternal and neonatal complications in preeclamptic women: A cross-sectional study

**Published:** 2017-09

**Authors:** Maryam Asgharnia, Fariba Mirblouk, Soudabeh Kazemi, Davood Pourmarzi, Mina Mahdipour Keivani, Seyedeh Fatemeh Dalil Heirati

**Affiliations:** 1 *Reproductive Health Research Center, Guilan University of Medical Sciences, Rasht, Iran.*; 2 *Guilan University of Medical Sciences, Rasht, Iran.*

**Keywords:** Pregnancy, High-risk, Preeclampsia, Uric acid, Pregnancy complications

## Abstract

**Background:**

Preeclampsia is associated with maternal and neonatal complications. It has been indicated that increased uric acid might have a predictive role on preeclampsia.

**Objective:**

We aimed to investigate the relationship between the level of uric acid with maternal and neonatal complications in women with preeclampsia.

**Materials and Methods:**

In this cross-sectional study, 160 singleton preeclamptic women at more than 28 wk gestational age were included. Hemoglobin, hematocrit, platelet count, liver and uric acid tests, and maternal and neonatal complications were assessed. The severity of preeclampsia, placental abruption, preterm labor, thrombocytopenia, elevated alanine aminotransferase and aspartate aminotransferase (ALT and AST), HELLP syndrome, eclampsia and required hospitalization in the ICU was considered as the maternal complication. Fetal complications were: small for gestational age (SGA), intrauterine fetal death, hospitalization in the neonatal intensive care unit, and Apgar score <7 at five minutes.

**Results:**

Of our participants, 38 women had severe preeclampsia (23.8%). The mean level of uric acid in women with severe preeclampsia was significantly higher than non-severe preeclampsia (p=0.031), also in those with an abnormal liver test (p=0.009). The mean level of uric acid in women with preterm delivery was significantly higher than women with term delivery (p=0.0001). Also, the level of uric acid had no effect on neonatal hospitalization in neonate invasive care unit. Based on logistic regression, the incidence of severe preeclampsia not affected by decreased or increased serum levels of uric acid.

**Conclusion:**

With higher level of uric acid in server preeclampsia we can expected more complications such as hepatic dysfunction and preterm delivery. Thus serum uric acid measurement can be helpful marker for severe preeclampsia.

## Introduction

Preeclampsia with the prevalence of 10.5% is a blood pressure disorder. It is a multi-systemic disease with unknown etiology. It is indicated by >140/90 mm Hg blood pressure with or without proteinuria after 20 wk of gestation. Hypertension during pregnancy is associated with maternal and neonatal complications (1, 2). In developed countries, 16% of maternal deaths are caused by high blood pressure which is higher than other factors such as bleeding (13%), abortion (8%) and sepsis (2%) (3). In Iran, the prevalence of hypertensive disorders during pregnancy is reported as 8.1% (4, 5). Up to now, there is no reliable, valid, and cost-effective screening test for the early detection of preeclampsia. It seems that measuring the blood level of uric acid is one of the available and cheap screening tests that have already been taken into account. Uric acid is the final product of purine metabolism oxidation which is mainly excreted in the urine. Since increased uric acid is the first and earliest laboratory presentation of preeclampsia as a result of reduced clearance of uric acid due to decreased glomerular filtration clearance, increased tubular reabsorption, and reduced secretion, some investigators use serum uric acid as a criterion for preeclampsia (3).

It has been indicated that uric acid might not only have a predictive role in preeclampsia but also play a significant role in the maternal and fetal pathogenesis and presentations (3). However, previous studies mentioned inconsistent results (6, 7). Some previous investigations mentioned a higher level of uric acid in patients with preeclampsia compared to healthy subjects (8, 9). However, other studies mentioned that changes in uric acid levels in preeclampsia might be as a result of associated diseases. They noted no important predictive value for uric acid (10, 11). Also, epidemiological studies on non-pregnant individuals showed that hyperuremia could be associated with hypertension, metabolic syndrome, coronary artery disease, cardiovascular disease and chronic kidney disease (12, 13).

Given the importance of preeclampsia in maternal and neonatal complications, in this study, we aimed to investigate the relationship between the level of uric acid with maternal and neonatal complications in women with preeclampsia. Knowing the effect of uric acid on the severity of preeclampsia and maternal and neonatal complications can help early diagnosis, control, and treatment. Results can be used for clinical decision-making and prevent from maternal and neonatal complications.

## Materials and methods

In this cross-sectional study, 160 preeclamptic women (severe and nonsevere) at more than 28 weeks gestational age admitted to Al-Zahra Hospital in Rasht, Iran during 2014-2015 were included. Diagnostic criteria were having hypertension to the extent of 140/90 mmgHg or more after 20 wk of pregnancy in women with or without proteinuria in previously normotensive. Also, hypertension in pregnant women with a history of preeclampsia was noted by >30 mm Hg increase in systolic blood pressure or >15 mmHg increase in diastolic blood pressure. Severe preeclampsia was diagnosed when systolic pressure ≥160 mmHg, diastolic pressure ≥110 mmHg with or without proteinuria, headache, visual changes, upper abdominal pain, oliguria, increased serum creatinine and liver transaminases and thrombocytopenia (<100,000). 

Measurement of blood pressure was performed by a single resident of obstetrics and gynecology. All pregnant women with smoking, diabetes or gestational diabetes, hemoglobinopathies, nephropathy, acute and chronic renal failure, glomerulopathy, urinary tract infection, hepatic dysfunction, gout, hypothyroidism or hyperthyroidism, fetal and placental malformations, and multiple pregnancies were excluded.

Of all women who met the inclusion criteria, 5 ml of blood from an anterior Cubital vein was taken and hemoglobin, hematocrit, platelet count, and liver and uric acid tests were assessed. For all women, 24-hr urine protein, creatinine, urine dipstick tests, and random urine samples were also performed. Uric acid was assessed using Pars test kit and by BT2000 (Italy) auto-analyzer system. All laboratory tests were conducted in Al-Zahra hospital.

Data including age, gravidity, parity, gestational age, and history of hypertension before pregnancy were collected through interviews. Weight and height were measured and body mass index (BMI) was calculated. Maternal complications included severity of preeclampsia, placental abruption, preterm labor (PTL), thrombocytopenia, elevated alanine aminotransferase (ALT) and aspartate aminotransferase (AST), HELLP syndrome (Hemolysis, Elevated Liver enzymes, and Low Platelet count), eclampsia and required hospitalization in the ICU were assessed. Neonatal complications including small for gestational age (SGA), intrauterine fetal death, hospitalization in the neonatal intensive care unit, and Apgar score <7 at five minutes were investigated.

**Ethical consideration**

This study was approved by the Ethics Committee of Guilan University of Medical Sciences (1930596730). Written informed consents were obtained from all participants before enrollment.

**Statistical analysis**

Data were analyzed using Statistical Package for the Social Sciences (version 21.0, SPSS Inc, Chicago, and Illinios, USA). Independent samples *t*-test was used to compare means between groups. Also, univariate logistic regression analysis using forward: LR method was used to assess the relationship between levels of uric acid and the incidence of maternal and neonatal complications. In the regression analysis, the effect of uric acid levels, age, gestational age, gravidity, parity, the platelet count, albuminuria, hemoglobin, hematocrit, creatinine, body mass index, and effect of hypertension before pregnancy on maternal and neonatal complications were examined. The p<0.05 indicated statistical significance and 95% confidence interval was noted.

## Results

In this study, 160 preeclamptic women participated, which 38 of them were severe preeclampsia (23.8%) and 122 of them were none severe (76.3%). The mean age of participants was 31.18±5.41 yr. 14.4% (n=23) had a history of hypertension before pregnancy and 71.3% (114) had albuminuria. Demographic characteristics and laboratory results have been reported in table I. Mean level of uric acid in women with severe pre- eclampsia (5.66±1.46) was significantly higher than non-severe preeclampsia (5.12±1.29) (p=0.031). In univariate analysis, the mean level of uric acid in women with hepatic dysfunction was significantly higher than the non hepatic dysfunction group (p=0.009). Also, women with preterm delivery had higher uric acid levels than women with normal term pregnancy (p=0.0001). Uric acid levels in women with neonates hospitalized in Neonate invasive care unit (NICU) were significantly higher than other women (p=0.016). In the case of maternal and neonatal complications, there was no significant difference between groups regarding the level of uric acid (Table II).

Based on the logistic regression analysis, 1 mg/dl increase in blood uric acid level induced 1.74 fold increase in the risk of hepatic dysfunction (OR: 1.74, 95% CI: 1.12-2.72). Also, with 1% increase in hematocrit, the chance of hepatic dysfunction will be about 1.3 times higher. Other variables had no effect on increasing or decreasing the risk of hepatic dysfunction in pregnant women with preeclampsia. In the present study by 1 mg/dl, increase in serum uric acid, 1.54 fold increase in the risk of PTL was noted (OR: 1.54, 95% CI: 1.15-2 ). Furthermore, by 1 mg/dl increase in serum hemoglobin and creatinin, 1.6 and 1.06 fold increase in the risk of PTL was noted, respectively. While other variables did not increase or decrease the chance of PTL in pregnant women with preeclampsia. 

Based on regression analysis, results showed that the level of uric acid had no effect on hospitalization of infants in the NICU. Also, the level of uric acid had no effect on neonatal hospitalization in NICU. However, by 1 mg/dl increase in hemoglobin and AST in pregnant women with preeclampsia, 1.6 and 1.022 fold increase in the risk of hospitalization in NICU was noted, respectively. The positive history of hypertension before pregnancy indicated 3.8 fold increase in hospitalization in NICU. (Table III).

**Table I:** Demographic characteristics and laboratory results of the study participants

**Variables**	**Mean±SD**
Age (yr)	31.18 ± 5.41
BMI (kg/m^2^)	32.33 ± 3.72
Gestational age (wk)	34.74 ± 2.64
Parity (n)	2.03 ± 1.10
Gravidity (n)	0.74 ± 0.94
Systolic blood pressure (mmHg)	152.19 ± 12.55
Diastolic blood pressure (mmHg)	98.19 ± 8.57
Platelets (/cumm)	201681 ± 56180
Hemoglobin (g/l)	12.33 ± 1.09
Hematocrit (%)	37.25 ± 3.19
AST (U/L)	32.35 ± 31.72
ALT (U/L)	26.64 ± 39.63
Uric acid (mmol/l)	5.25 ± 1.35
Creatinine (mg/dl)	0.76 ± 0.15

BMI: Body mass index

ALT: alanine aminotransferase

AST: aspartate aminotransferase

**Table II:** Univariate analysis of pregnancy outcomes in study participants

**Maternal and fetal complications**		**Number**	**Uric acid level**	**p-value**
Placental abruption				
	Yes	1	6.40	0.393
	No	159	5.24± 1.38	
Thrombocytopenia				
	Yes	4	4.20 ±0.86	0.115
	No	156	5.28 ± 1.35	
Hepatic dysfunction				
	Yes	11	6.27 ± 1.40	0.009
	No	149	5.17 ± 1.32	
ICU hospitalization (maternal)				
	Yes	17	5.61± 1.67	0.241
	No	143	5.21 ±1.30	
HELLP syndrome				
	Yes	3	6.20 ± 2.27	0.218
	No	157	5.23 ± 1.33	
PTL				
	Yes	79	5.63 ± 1.38	0.0001
	No	81	4.87 ± 1.21	
IUFD				
	Yes	4	5.90 ± 1.17	0.329
	No	156	5.23 ± 1.35	
SGA				
	Yes	30	5.61± 1.39	0.103
	No	130	5.16 ± 1.33	
Apgar score <7 at five minute				
	Yes	4	4.50± 2.10	0.278
	No	151	5.24 ± 1.33	
Need to NICU				
	Yes	32	5.74± 1.37	0.016
	No	124	5.10 ± 1.32	

Data presented as mean±SD. Independent *t*-test

NICU: Neonate invasive care unit

PTL: Preterm labor

IUFD: Intrauterine fetal death

SGA: Small gestational age

**Table III:** Logistic regression analysis on the factors affecting the incidence of hepatic dysfunction, premature birth, and NICU hospitalization in women with preeclampsia

**Complication**		**Regression coefficient**	**S.E**	**OR**	**95% CI**		**p-value**
					**Lower bound**	**Upper bound**	
Hepatic dysfunction							
	Level of uric acid	0.554	0.227	1.740	1.115	2.176	0.015
	Level of hematocrit	0.270	0.111	1.311	1.054	1.630	0.015
Premature birth							
	Level of uric acid	0.435	0.151	1.544	1.148	2.077	0.004
	Level of hemoglobin	0.477	0.176	1.611	1.142	2.272	0.007
	Level of creatinin	0.062	0.018	1.064	1.026	1.120	0.001
NICU hospitalization							
	Level of hemoglobin	0.455	0.216	1.576	1.032	2.407	0.035
	AST	0.022	0.009	1.022	1.004	1.040	0.015
	HTN before pregnancy	1.323	0.523	3.753	1.347	1.457	0.011

Logistic regression

NICU: Neonate invasive care unit

SE: Standard error

HTN: Hypertension

OR: Odds ratio

CI: Confidence interval

AST: aspartate aminotransferase

## Discussion

In this study, the mean level of uric acid in women with severe preeclampsia was significantly higher than non-severe preeclampsia. In a study by Sultana and colleagues, which compared the mean levels of uric acid in subjects with normal blood pressure and preeclampsia, results showed that preeclampsia was associated with hyperuricemia (14). Also, Gianni *et al *followed patients for a month after delivery and mentioned uric acid as a reliable predictor of preeclampsia in women with gestational hypertension. They indicated uric acid with cut-off of 309 µm/l as a predictor of preeclampsia (8).

Enaruna *et al* also showed that uric acid levels in women with severe pre-eclampsia were higher than the control group (11). Chen and co-workers showed a higher level of serum uric acid in women with preeclampsia and the increase occurred with the onset of preeclampsia. There was no significant difference between preeclampsia and normal subjects regarding the mean level of uric acid in the first and second trimesters. Chen *et al* stated that serum uric acid levels increased with the onset of clinical signs of preeclampsia, but it might not be a predictor of preeclampsia and should not be considered as a predictive biomarker (15). According to results, although preeclampsia might cause an increase in uric acid, the inverse association did not exist. Laughon *et al *mentioned that the increase of uric acid in the first trimester of pregnancy was associated with preeclampsia and hypertension during pregnancy was related to hyperuricemia (16). 

Hawkins *et al* reported that hyperuricemia in pregnant women with high blood pressure was an important finding which might expose women to adverse maternal and fetal complications. Even women with gestational hypertension, without other signs of pre-eclampsia had adverse embryonic complications such as SGA and pre-maturity. In this study, uric acid was measured near-term period which was similar to ours (17).

Elmas *et al* showed higher levels of uric acid, xanthine oxidase and Allantoin activity and blood pressure in patients with preeclampsia versus the control group (18). Elmas *et al* suggested that increased uric acid, xanthine oxidase and Allantoin activity might be noted following the death of placental cells consequent the abnormal activity of trophoblast in preeclampsia. Therefore, currently, clinicians prefer the mentioned causes for increased uric acid in preeclampsia instead of the effect of kidney disease following preeclampsia (19).

Corominas *et al* showed that up to 20 weeks of gestation, the mean level of uric acid in preeclampsia and normal women were similar. But after 20^th^ wk of gestation, the mean level of uric acid in women with preeclampsia was 1.5 fold higher than control and healthy subjects. This increase coincided with stable creatinin and urea in patients and showed increased uric acid should be separated from kidney disorders (20). However, Zhao *et al* associated increased uric acid in women with preeclampsia with the systemic inflammation of maternal blood vessels, which could be presented by increased Tumor necrosis factor alpha TNF-alpha and Intercellular adhesion molecule- 1 (21). Matias *et al* as also expressed the increased uric acid by inflammatory reactions, and this phenomenon was associated with that expression of nod-like intracellular receptors with a pyrin domain NLRP3 gene in pregnant women with preeclampsia (22).

Previous studies showed a significant association between hyperuricemia and adverse obstetric outcomes in pregnant women with hypertension (23, 24) which was similar to the current study. As in this study higher mean level of uric acid was associated with increased rate of PTL and consequently NICU hospitalization. In a study by James *et al* results showed that women with hypertension and hyperuricemia had a similar or higher risk comparing women with high blood pressure and proteinuria. Risks included a shorter period of pregnancy, lower percentiles of birth weight, increased risk of preterm birth and SGA. They concluded that hyperuricemia was at least as effective as proteinuria to identify pregnancies with the risk of high blood pressure (25).

In this study, although uric acid levels in women who were hospitalized in the NICU was higher than non-hospitalized women, this association was not significant. While Andrews *et al* which assessed Indian patients with high blood pressure for two years showed that the rate of maternal death in those with increased uric acid levels, LDL, AST, alanine aminotransferase, APTT was higher than the control group (26). In the present study, although the incidence of neonatal complications such as intrauterine fetal death, low Apgar score and SGA in preeclamptic women was slightly higher than the control group, this relation was not significant. While Patel et al which compared levels of uric acid (<6 and ≥6) in women with high blood pressure Indicated hyperuricemia in patients with high blood pressure as a risk factor for several serious maternal and perinatal complications such as <7 Apgar score, intrauterine death, eclampsia, and cesarean delivery in patients with ≥6 level of uric acid versus <6 (27).

Results showed that by 1 mg/dl increase in serum uric acid and hematocrit in pregnant women with preeclampsia, 1.7 and 1.3 folds increases in the risk of hepatic dysfunction were noted, respectively. In addition, by 1 mg/dl increase in serum uric acid and hemoglobin, 1.5 and 1.6 fold increases in the risk of hepatic dysfunction were noted, respectively. In a study by Hawkins *et al*, with increasing levels of uric acid, the risk of kidney failure which could be diagnosed by the increased level of creatinin in the mothers was four times higher compared with the control group (17).

In the present study by 1 mg/dl increase in serum hemoglobin and creatinine, 1.6 and 1.06 fold increases in the risk of PTL was noted, respectively. While other variables did not increase or decrease the chance of PTL in pregnant women with preeclampsia. Also, the level of uric acid had no effect on neonatal hospitalization in NICU. However, by 1 mg/dl increase in hemoglobin and AST in pregnant women with preeclampsia, 1.6 and 1.022 fold increases in the risk of neonatal hospitalization in NICU were noted, respectively. 

The positive history of hypertension before pregnancy indicated 3.8 fold increase in neonatal hospitalization in NICU. Livingston et al stated that level of uric acid was associated with prenatal and maternal complications in women with preeclampsia (3). Wu *et al* also declared that with one increase in standard deviation of serum uric acid, 1.5 fold increases in the chance of maternal and fetal complications were noted (28). In this study, uric acid was higher in severe preeclampsia and PTL. Whereas in severe preeclampsia, clinicians ought to terminate the pregnancy to save the patient’s life, the higher rate of PTL would be expected. 

## Conclusion

In this study, we found a significant relationship between the level of uric acid with hepatic dysfunction and PTL in pregnant women with preeclampsia. As assessing serum uric acid in the laboratory can be easily performed, it seems that it might be diagnostic and prognostic criteria for determining the early complications of preeclampsia and can be used to treat and manage women with preeclampsia.

## Note

***This article extracted from M.D. thesis. (Mina Mahdipour Keivani)***
